# Debridement, antimicrobial therapy, and implant retention (DAIR) as curative surgical strategy for acute periprosthetic hip and knee infections: a summary of the position paper from the European Bone & Joint Infection Society (EBJIS)

**DOI:** 10.5194/jbji-10-139-2025

**Published:** 2025-04-01

**Authors:** Irene K. Sigmund, Marjan Wouthuyzen-Bakker, Tristan Ferry, Willem-Jan Metsemakers, Martin Clauss, Alex Soriano, Rihard Trebse, Ricardo Sousa

**Affiliations:** 1 Department of Orthopaedics and Traumatology, Medical University of Vienna, Vienna, Austria; 2 Department of Medical Microbiology and Infection Prevention, University Medical Center Groningen, University of Groningen, Groningen, the Netherlands; 3 Infectious and Tropical Diseases Unit, Croix-Rousse Hospital, Hospices Civils de Lyon, Lyon, France; 4 Department of Trauma Surgery, University Hospitals Leuven, Leuven, Belgium; 5 Department of Development and Regeneration, KU Leuven, Leuven, Belgium; 6 Department of Orthopaedics and Trauma Surgery, Center for Musculoskeletal Infections (ZMSI), University Hospital Basel, Basel, Switzerland; 7 Hospital Clinic of Barcelona, Barcelona, Spain; 8 IDIBAPS, Barcelona, Spain; 9 CIBERNIF, CIBER in Infectious Diseases, ISCIII, Madrid, Spain; 10 Orthopaedic Hospital Valdoltra, Ankaran, Slovenia; 11 Orthopaedic Department, Porto Bone Infection Group (GRIP), ULS Santo António, Porto, Portugal

## Abstract

This is a summary of our position paper on debridement, antimicrobial therapy, and implant retention (DAIR) procedures as curative treatment strategy for acute periprosthetic hip and knee infections. It includes the defined indications as well as the contraindications for DAIR procedures when eradication/cure is intended, based on the currently available literature. Risk factors which need to be considered during the decision-making process are described. Additionally, we give an overview of important surgical and medical considerations in the management of acute PJI patients treated with DAIR.

## 1 Introduction

Debridement, antimicrobial therapy, and implant retention (DAIR) is one of the surgical treatment options for periprosthetic joint infection (PJI). When infection eradication is the primary goal, patient selection and adequate surgical and medical management are of utmost importance. In the last decade, multiple factors have been shown to be associated with DAIR failure or success. It is, therefore, necessary to identify patients who benefit from this treatment modality.

In this summary of our position paper, we provide recommendations based on the most robust and most recently published data (Sigmund et al., 2025). It is intended for use by clinicians in daily practice (orthopaedic surgeons, infectious diseases specialists, microbiologists, and other healthcare professionals caring for PJI patients) to improve overall results and patient care.

## Indications

In general, a DAIR procedure should be considered in patients with (Sigmund et al., 2025)

1. a well-fixed, well-positioned, and well-functioning prosthesis;
2. an acute infection including early acute ( ≤4 weeks after index arthroplasty) and late acute infections ( <3 weeks of symptoms after an uneventful postoperative period and >4 weeks after index arthroplasty); and
3. good conditions of the surrounding soft tissue without a sinus tract.

Optimally, the microorganism and its susceptibility to the antimicrobial therapy are known prior to the procedure. However, further management (DAIR) should not be delayed in all cases until microbiological results are available as symptom onset of >3 weeks can reduce the success rate as well (Sigmund et al., 2025).

## Contraindications

A DAIR procedure is not recommended as a curative surgical strategy in patients with (Sigmund et al., 2025)

1. a loose prosthesis;
2.
>12 weeks after index arthroplasty;
3.
>3 weeks of symptoms;
4. compromised soft tissues (primary closure impossible); and/or
5. a sinus tract.

In cases fulfilling the above-mentioned criteria, the chances of a successful DAIR procedure and infection eradication are low. An exchange of the whole implant should be considered.

## Other risk factors to consider

1. Patients with an early acute infection between 4–12 weeks after index arthroplasty may have a higher risk of DAIR failure.
2. Patients with previous revision surgery(ies) showed higher failure rates following DAIR in comparison to patients with a DAIR procedure after primary arthroplasty in some studies.
3. Some host and clinical factors were described as independent predictors of DAIR failure in some studies: patients with rheumatoid arthritis, chronic obstructive pulmonary disease (COPD), and/or immunosuppressive therapy were associated with worse outcomes after DAIR in some studies.
4. Some large multicentre studies reported worse outcomes in *Staphylococcus aureus* infections (especially in late acute PJIs).
5. An infection caused by difficult-to-treat microorganism(s) (defined as microorganism where no biofilm active antimicrobial therapy is available) and/or fungal infections may have a higher risk of DAIR failure.
6. The presence of bacteraemia in acute infections was associated with worse outcomes in the literature.

These risk factors are associated with a potentially higher failure rate following DAIR but are not considered contraindications (Sigmund et al., 2025). Due to the available results, all factors listed above should be considered in the decision-making process prior to the surgical treatment. In these cases, an individual multidisciplinary team discussion considering all risk factors is recommended to find the optimal/individual treatment option.

## Surgical considerations

1. A thorough and adequate debridement is of paramount importance and should be performed in an open procedure. Arthroscopic washouts were associated with inferior outcomes.
2. Due to improved results in the literature (Sigmund et al., 2025), modular components should be exchanged whenever possible to ensure the best chance of DAIR success. A better visualization and accessibility to the joint for the debridement is then guaranteed, probably leading to an improved bioburden reduction.
3. A standardized tissue sampling including four to six deep tissue samples for microbiology and histology is recommended.
4. A copious lavage with saline (and antiseptic solutions) should be performed.

In general, DAIR is not an emergency that needs to be performed within 24 h. However, it should be performed in a timely manner within 3 weeks (preferably within the first 7 d) of symptoms, once the patient is optimized, modular components for exchange are available, and preferably by an experienced surgical team (Sigmund et al., 2025).

**Table 1:**
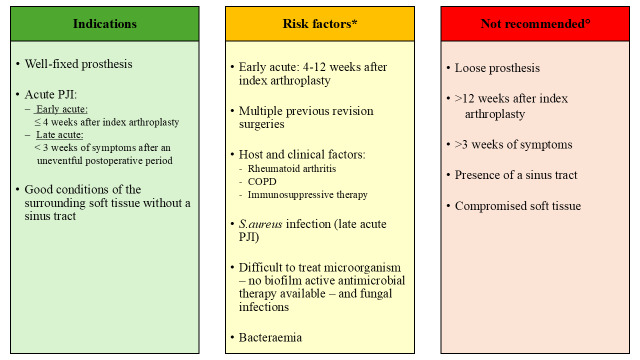
Indications, risk factors to consider in the decision-making process, and contraindications for a DAIR (debridement, antimicrobial therapy, and implant retention) procedure in periprosthetic hip and knee infections when cure is intended based on the current literature.

^*^ These host and clinical factors can be associated with a higher risk of failure. ° If cure is intended, in patients fulfilling these factors, an exchange of the whole implant should be considered.

## Medical considerations

1. In patients without sepsis or septic shock, antimicrobial treatment should be withheld until tissue sampling is completed (Sigmund et al., 2025).
2. A total antibiotic duration of 12 weeks after DAIR involving an induction period of 1 week of intravenous (IV) treatment is currently recommended (Sigmund et al., 2025).
3. Broad-spectrum antibiotic regimes should be adjusted according to the antibiogram once microbiological results are available (Sigmund et al., 2025).
4. A combination of fluoroquinolones with rifampicin is recommended for staphylococcal infections and fluoroquinolones for Gram negatives (Sigmund et al., 2025).
5. Rifampicin can be started once source control has been achieved, based on clinical and laboratory parameters (Sigmund et al., 2025).

In Table 1, the indications, contraindications, and other risk factors for a DAIR procedure are listed. These recommendations can be applied when eradication/cure of PJI is intended. For final decision making, it is advisable that patients are discussed in a multidisciplinary team (orthopaedic surgeon, infectious diseases physician, microbiologist, radiologist) on a case-by-case basis. With careful patient selection, a dedicated multidisciplinary team, and an adequate surgical technique, reasonable outcomes after a DAIR procedure can be achieved.

This summary is the product of a very comprehensive literature review, which can be found in the EBJIS position paper on DAIR (Sigmund et al., 2025).

## Data Availability

In this summary of our position paper, we provide recommendations based on the most robust and recently published data (Sigmund et al., 2025).
